# Phase I and pharmacokinetic study of the combination of topotecan and ifosfamide administered intravenously every 3 weeks

**DOI:** 10.1038/sj.bjc.6601861

**Published:** 2004-05-11

**Authors:** T Kerbusch, G Groenewegen, R A A Mathôt, V M M Herben, W W ten Bokkel Huinink, M Swart, B Ambaum, H Rosing, S Jansen, E E Voest, J H Beijnen, J H M Schellens

**Affiliations:** 1 Department of Pharmacy and Pharmacology, The Netherlands Cancer Institute/Slotervaart Hospital, Amsterdam, The Netherlands; 2 Department of Medical Oncology, The Netherlands Cancer Institute/Antoni van Leeuwenhoek Hospital, Amsterdam, The Netherlands; 3 Department of Medical Oncology, University Medical Center Utrecht, The Netherlands; 4 Faculty of Pharmaceutical sciences, University of Utrecht, The Netherlands

**Keywords:** phase I, pharmacokinetics, pharmacodynamics, topotecan, ifosfamide

## Abstract

To determine the maximum-tolerated dose (MTD), dose-limiting toxicities, and pharmacokinetics of topotecan administered as a 30-min intravenous (i.v.) infusion over 5 days in combination with a 1-h i.v. infusion of ifosfamide (IF) for 3 consecutive days every 3 weeks. Patients with advanced malignancies refractory to standard therapy were entered into the study. The starting dose of topotecan was 0.4 mg m^−2^ day^−1^ × 5 days. Ifosfamide was administered at a fixed dose of 1.2 g m^−2^ day^−1^ × 3 days. In all, 36 patients received 144 treatment courses. Owing to toxicities, the schedule of topotecan administration was reduced from 5 to 3 days. The MTD was reached at topotecan 1.2 mg m^−2^ day^−1^ × 3 days with IF 1.2 g m^−2^ day^−1^ × 3 days. Haematological toxicities were dose limiting. Neutropenia was the major toxicity. Thrombocytopenia and anaemia were rare. Nonhaematological toxicities were relatively mild. Partial responses were documented in three patients with ovarian cancer dosed below the MTD. Topotecan and IF did not appear to interact pharmacokinetically. The relationships between the exposure to topotecan lactone and total topotecan, and the decrease in absolute neutrophil count and the decrease in thrombocytes, were described with sigmoidal–*E*_max_ models. The combination of 1.0 mg m^−2^ day^−1^ topotecan administered as a 30-min i.v. infusion daily times three with 1.2 g m^−2^ day^−1^ IF administered as a 1-h i.v. infusion daily times three every 3 weeks was feasible. However, the combination schedule of topotecan and IF did result in considerable haematological toxicity and in conjunction with previously reported pronounced nonhaematological toxicities and treatment related deaths, it may be concluded that this is not a favourable combination.

Topotecan (Hycamtin®) is a semisynthetic analogue of the alkaloid camptothecin that acts as a specific inhibitor of the nuclear enzyme topoisomerase-I (Herben *et al*, 1996). Inhibition of this enzyme results in lethal DNA damage during transcription and replication. The E-ring lactone in the basic structure of topotecan is considered essential for the interaction with topoisomerase-I (Herben *et al*, 1996) *In vivo*, rapid conversion of the active lactone to the inactive carboxylate form occurs. Topotecan is indicated for second-line treatment of advanced ovarian cancer or refractory small-cell lung cancer, and has demonstrated antitumour activity in non-small-cell lung cancer, prostate cancer, and colorectal cancer (Herben *et al*, 1996).

Ifosfamide (IF) (Holoxan®, Ifex®) is an alkylating agent, which is indicated as third-line agent in the treatment of germ cell testicular cancer in combination with other chemotherapeutic agents. It is also used to treat cancers of the head and neck, breast, cervix, ovaries, soft tissue sarcoma, Ewing's sarcoma, osteosarcoma, both Hodgkin's and non-Hodgkin's lymphomas, non-small-cell lung cancer, acute lymphocytic leukaemia, and neuroblastoma (Kerbusch *et al*, 2001a). Ifosfamide is a prodrug, which needs to undergo activation by cytochrome *P*450-3A4 (CYP3A4) to 4-hydroxyifosfamide (4OHIF). Intracellular spontaneous decomposition of 4OHIF yields the ultimate alkylating metabolite ifosforamide mustard (IFM). Ifosfamide is deactivated to the noncytotoxic metabolites 2- and 3-dechloroethylifosfamide (2DCE and 3DCE), yielding an equimolar amount of neurotoxic chloroacetaldehyde (Cerny and Küpfer, 1992). Ifosfamide metabolism is autoinducible (Kerbusch *et al*, 2000b).

In a phase I study with the combination topotecan and cyclophosphamide, an isomer of IF, synergism was suggested between alkylating agents and topoisomerase-I inhibitors (Murren *et al*, 1997). It was proposed that DNA alkylation triggers DNA repair mechanisms, which may depend on topoisomerase-I. Ifosfamide was chosen as representative alkylator because it is associated with less haematological toxicity than cyclophosphamide (Kerbusch *et al*, 2001a).

These data led us to evaluate a chemotherapeutic regimen of topotecan in combination with IF. Aims of this phase I trial were to determine the maximum-tolerated dose (MTD), dose-limiting toxicity (DLT), and the safety profile of the combination of daily 30-min infusions of topotecan on days 1–5 followed by 1.2 g m^−2^ IF administered as a 1-h infusion daily on days 1–3 every 3 weeks in patients with advanced malignancies. Secondary aims were the assessment of the pharmacokinetics of topotecan and IF and its main metabolites when given in combination and documentation of any antitumour activity of this regimen.

## PATIENTS AND METHODS

### Eligibility criteria

The eligibility criteria were histologically confirmed metastatic or locally advanced cancer, no proven benefit of existing anticancer therapy, age between 18 and 75 years, World Health Organisation (WHO, 1979) performance status ⩽2, adequate bone marrow function (white blood cells (WBC) ⩾3.5 × 10^9^ l^−1^, granulocytes ⩾1.5 × 10^9^ l^−1^, platelets ⩾100 × 10^9^ l^−1^, haemoglobin ⩾6 mmol l^−1^), adequate hepatic function (serum bilirubin ⩽20 *μ*mol l^−1^, alanine-aminotransferase (ALAT), aspartate-aminotransferase (ASAT), and alkaline phosphatase (AP) ⩽2 × the upper limit of the reference range or ⩽5 × the upper limit of the reference range if the elevation was directly contributed to the presence of metastatic disease), adequate renal function (serum creatinine ⩽120 *μ*mol l^−1^ or creatinine clearance (CL) ⩾60 ml min^−1^), evaluable disease, and a life expectancy ⩾3 months. The exclusion criteria were breast feeding, pregnancy or inadequate contraceptive protection, drug, medication or alcohol abuse, treatment with an investigational drug ⩽1 month prior to the study entry, surgery, radiotherapy (except for analgesic indications) or chemotherapy (mitomycin C or nitrosoureas ⩽1.5) ⩽1 month prior to the study entry, other diseases altering procedures of the trial, history of seizures or central nervous system disorders, severe intercurrent infection, serious uncontrolled concurrent medical or psychiatric disease, known hypersensitivity to IF or topotecan, and prior treatment with IF or topotecan. The study protocol was approved by the Ethics Review Boards of the Antoni van Leeuwenhoek Hospital/The Netherlands Cancer Institute and University Medical Center Utrecht. The trial was conducted in these two centres according to Good Clinical Practice and the current edition of the Declaration of Helsinki. Written informed consent was obtained from all patients entering the study.

### Toxicity and response evaluation

Pretreatment evaluation included a complete medical history and complete physical examination. Before each course, blood chemistry and haematological profiles were checked, and urinalysis was performed. Complete blood cell counts and blood chemistry were repeated weekly. Electrocardiograms and tumour measurements (by physical examination, chest X-ray, other radiological investigations or ultrasound) were performed every other cycle. All toxicities were graded according to the National Cancer Institute Common Toxicity Criteria (NCI-CTC, 1998). Dose-limiting toxicities were defined as any of the following events occurring during the first treatment cycle and attributable to either IF or topotecan: (1) grade IV neutropenia lasting ⩾7 days or of any duration associated with severe systemic infection requiring parental treatment; (2) febrile neutropenia; (3) grade III or IV thrombocytopenia with or without haemorrhagic complications; or (4) grade III or IV nonhaematological toxicity excluding nausea/vomiting responsive to treatment, anorexia, and alopecia. The MTD was defined as the dose at which ⩾two out of three or ⩾two out of six patients experienced DLT. The next lower dose level below the MTD was the recommended dose for phase II studies. Responses were determined according to the WHO criteria (WHO, 1979).

### Dose escalation

Doses of topotecan were escalated during the study. Starting dose was 0.4 mg m^−2^ day^−1^ topotecan for 5 days by means of a 30-min infusion. Escalation was planned with 0.2 mg m^−2^ day^−1^ increments per dose level. Ifosfamide was administered at a fixed dose of 1.2 g m^−2^ day^−1^ for 3 days by means of a 1-h infusion. Ifosfamide was administered directly after the topotecan infusion. Upon identification of the MTD, the protocol was amended to reduce topotecan dosing from 5 to 3 days. Treatment cycles were repeated every 21 days, provided patients had sufficiently recovered from any drug-related toxicity associated with the previous course (nonhaematological toxicity ⩾grade I, alopecia excluded, haemoglobin ⩾6 mmol l^−1^ and return of blood cell counts to ⩾1.5 × 10^9^ l^−1^ neutrophils and ⩾100 × 10^9^ l^−1^ platelets). Dose modifications of both IF and topotecan were made if poor subjective tolerance to treatment occurred. Neutrophils <0.5 × 10^9^ l^−1^ associated with temperature ⩾38.5°C or lasting longer ⩾7 days, neutrophils <1.0 × 10^9^ l^−1^ lasting longer ⩾21 days, platelets <50 × 10^9^ l^−1^ or any nonhaematological toxicity requiring a delay in treatment resulted in dose reduction of 25% and platelets <25 × 10^9^ l^−1^ resulted in dose reduction of 50%.

### Drug administration

Topotecan (Hycamtin®) was supplied by GlaxoSmithKline (Zeist, The Netherlands) in vials containing topotecan HCl, equivalent to 5 mg of free base. Inactive ingredients were 60 mg mannitol and 25 mg tartaric acid. The content of each vial was reconstituted with 2 ml sterile water for injection. The appropriate dosage of the drug was diluted in 50 ml of 0.9% NaCl solution. Topotecan was administered through a peripheral venous access device using a syringe pump. Ifosfamide (Holoxan®) was supplied by Asta Medica BV (Diemen, The Netherlands) in vials containing 0.5, 1 or 2 g without excipients. Ifosfamide was diluted in 0.9% NaCl to approximately 1 l and administered through a peripheral venous access device using an ambulatory infusion pump. Supportive care consisted of 5HT_3_-antagonists, corticosteroids and mesna. Mesna was administered as protective agent for IF-induced bladder toxicity. The bolus dose of mesna was 50% of the dose of IF, which was followed by continuous intravenous (i.v.) infusion of mesna at a dose of 50% of the dose of IF during and 8 h after the IF infusion. The protocol did not allow the use of bone marrow granulocyte colony-stimulating factors (G-CSF).

### Pharmacokinetic sampling

Pharmacokinetic studies were performed for topotecan (total and lactone form) and IF and metabolites (2- and 3DCE, 4OHIF, and IFM) during the first two treatment courses. Intravenous samples were drawn from an indwelling intravenous cannula placed in the arm contralateral to the arm receiving chemotherapy prior to the topotecan infusion and at the following time points after the start of the topotecan infusion: 15, 30 (prior to the end of the topotecan infusion), 60 and 90 min (prior to the end of the IF infusion), and 2, 3, 4.5, 7.5, 11, and 24 h (prior to the start of the following topotecan infusion). Pharmacokinetic sampling was executed according to this schedule on days 1 and 3 of cycle 1 and day 1 of cycle 2. Blood samples were immediately placed on icewater. The plasma was immediately separated by centrifugation at 1000 **g** for 5 min at 4°C. For the analysis of topotecan lactone plasma protein precipitation was performed by adding 1 ml plasma to 2 ml cold methanol (−20°C). The sample was mixed on a whirl mixer for 10 s and centrifuged for 3 min at 1000 **g** at 4°C. The clear supernatant was transferred to a polypropylene tube and immediately stored at −70°C pending analysis. Furthermore, plasma was aliquoted in three 1-ml volumes. Two aliquots were used for 4OHIF analysis after stabilisation with semicarbazide and one aliquot was used for IFM analysis after stabilisation with semicarbazide and sodium chloride. The remaining plasma was aliquoted to two 0.5-ml volumes for the analysis of IF, 2DCE and 3DCE, and total topotecan. Urine was collected during the first course for 96 h after the start of the IF infusion and an aliquot was analysed for IF, 2DCE and 3DCE. Both plasma and urine samples were immediately stored at −70°C, pending analysis. Total topotecan (lactone plus carboxylate form) and the lactone form were determined separately using a high-performance liquid chromatographic (HPLC) system with fluorometric detection (Rosing *et al*, 1995). Gas chromatography with selective nitrogen–phosphorous detection was used for the determination of IF, 2DCE and 3DCE, in urine and plasma (Kerbusch *et al*, 2000c). Two separate HPLC methods were used for the determination of 4OHIF and IFM concentrations (Kerbusch *et al*, 1998, 2000a).

### Pharmacokinetic evaluation

The pharmacokinetics of lactone and total topotecan were described with a two-compartment model, yielding CL (l h^−1^), volume of distribution of the central compartment (*V*, l), volume of distribution at steady state (*V*_ss_, l) and intercompartmental CL (*Q*, l h^−1^). The pharmacokinetics of IF and its main metabolites were described using an integrated nonlinear model with autoinduction (Kerbusch *et al*, 2000b, 2001b and 2001c). This model described the pharmacokinetics of IF with an initial CL of IF (CL_init_, l h^−1^) and a volume of distribution (*V*_ifo_, l), and autoinduction with a first-order rate constant for enzyme degradation/inactivation (*K*_enz,out_, h^−1^) and an IF concentration at 50% of the maximum inhibition of enzyme degradation (IC_50_, *μ*M). The induction half-life of the enzyme (*t*_1/2,enz_, h) was calculated by the ratio of ln(2) and *K*_enz,out_. The pharmacokinetics of the metabolites were described with a ratio of the fraction of IF metabolised and the volume of distribution of that metabolite (*F*_m_/*V*_m_=F^*^, l^−1^), an elimination rate constant (*K*, h^−1^) and a rate constant (F^**^) from 4OHIF to IFM (Kerbusch *et al*, 2001b). The exposure to a compound was described by the area under the plasma concentration–time curve (AUC). All pharmacokinetic models were fitted to the data from the individuals using the population pharmacokinetic program NONMEM (Non-linear Mixed Effects Modelling, Version V) (Boeckman *et al*, 1994), with first-order conditional estimation (FOCE). The residual or intraindividual variability of the pharmacokinetics were described separately with a combined proportional and additive term. The interindividual variability (IIV) of each pharmacokinetic parameter was estimated using a proportional error model.

### Pharmacokinetic–pharmacodynamic relationships

Relationships between the AUC of lactone and total topotecan and myelosuppression were explored using scatter plots of the AUC *vs* the percentage decrease in absolute neutrophil count (ANC) and thrombocytes (THR). The percentage decrease was defined as:


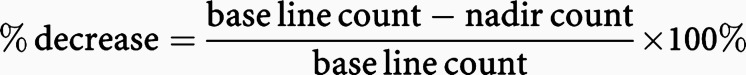


The data were fit to a sigmoidal maximum effect (*E*_max_) model, as described by the modified Hill equation (Holford and Sheiner, 1992):


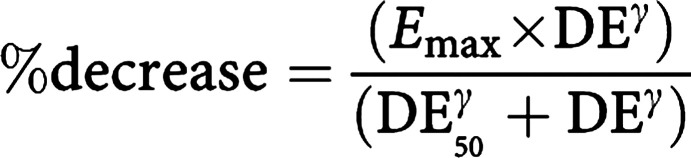


where *E*_max_ denotes the maximal effect that can be elicited, DE is a measure of drug exposure (ie AUC), DE_50_ represents the drug exposure associated with 50% of *E*_max_ and *γ* is the Hill coefficient, which describes the sigmoidicity of the curve. Since the percentage decrease could not exceed 100%, *E*_max_ was fixed to 100. Separate exposure–response models were described for total and lactone form of topotecan using NONMEM with FOCE and interaction. The residual variability (RV) was described with an additive term. The interoccasion variability (IOV) of AUC_50_ was estimated using a proportional error model. In addition, combined exposure–effect models (additive) were explored of the topotecan plus IF–myelosupression relationship. Additivity was modelled by assuming separate terms (Hill equations) for each active moiety (A and B) plus a scaling for relative potency (RP):


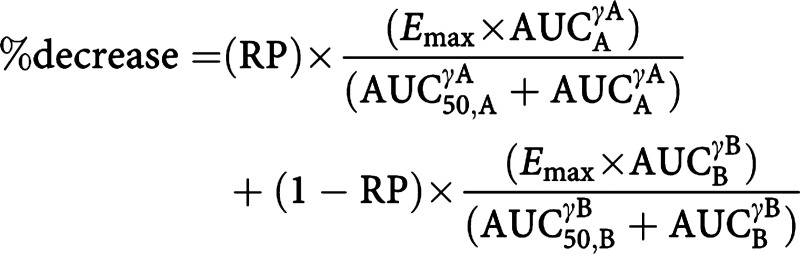


The active moieties (A and B) tested were topotecan lactone/total and 4OHIF.

## RESULTS

### Patients

At the end of the study, 36 patients received 144 courses at seven dose levels. Patient characteristics are listed in Table 1

**Table 1:** Patient characteristics

**Characteristic**	**Number**	**%**
Number of patients	36	
Male/female	6/30	
		
*Age (years)*		
Median	57	
Range	32–77	
		
*Performance status*		
0	12	33
1	20	56
2	4	11
		
*Primary tumour site*		
Colon	2	6
Cervix	1	3
Melanoma	1	3
Ovarian	22	61
Gastric	2	6
Pancreas	1	3
Renal	1	3
Oropharynx	1	3
Non-small-cell lung cancer	4	11
Urogenital	1	3
		
Prior palliative chemotherapy	34	94
*Number of previous regimens*		
Median	1	
Range	1–6	
Prior radiotherapy	7	19

. The medium number of courses administered per patient was 3 (range 1–10). In four patients only one treatment course was administered because of rapidly progressive disease. Two of these patients developed an ileus, one with a septic shock and one with lethal intestinal bleeding. This was considered disease related. Two courses were delayed in one patient and treatment discontinued in another at dose level 5 (1.0 mg m^−2^ day^−1^ × 3), due to the occurrence of an ileus.

### Dose escalation

All patients were assessable for toxicity during the first treatment course. Doses of topotecan were escalated according to the scheme depicted in Table 2

**Table 2:** Topotecan dose escalation

**Dose level**	**Number of patients**	**DLT type and grade**	**Topotecan dose**	**Total topotecan dose (mg m^−2^)**
1	3		0.4 mg m^−2^ day^−1^ × 5 days	2.0
2	3 (DLT in one of three)	T III, N IV, A IV, toxic death	0.6 mg m^−2^ day^−1^ × 5 days	3.0
1	2		0.4 mg m^−2^ day^−1^ × 5 days	2.0
3	6		0.6 mg m^−2^ day^−1^ × 3 days	1.8
4	3		0.8 mg m^−2^ day^−1^ × 3 days	2.4
5	3		1.0 mg m^−2^ day^−1^ × 3 days	3.0
6	6 (DLT in one of six)	N IV, fever⩾7 days	1.2 mg m^−2^ day^−1^ × 3 days	3.6
7	4 (DLT in two of four)	N IV, fever⩾7 days (both)	1.4 mg m^−2^ day^−1^ × 3 days	4.2
6	2 (DLT in one of two)	N IV, fever⩾7 days	1.2 mg m^−2^ day^−1^ × 3 days	3.6
5	4		1.0 mg m^−2^ day^−1^ × 3 days	3.0

When level 7 resulted in DLT additional patients were recruited at level 6 and subsequently at level 5. The latter is the advised level for further studies.

T=thrombocytopenia; N=neutropenia; A=anaemia; DLT=dose-limiting toxicity.

. Myelosuppression was the DLT for the combination of topotecan and IF in this schedule (Table 3

**Table 3:** Haematological toxicities in 144 courses

				**Anaemia**^a^		**Neutropenia**^a^		**Thrombocytopenia**^a^	
**Doselevel**	**Topotecan dose**	**Number of patients**	**Number of courses**	**Grade III**	**Grade IV**	**Grade III**	**Grade IV**	**Grade III**	**Grade IV**
1	0.4 mg m^−2^ × 5 days	5	20	0 (0%)/1 (5%)	0 (0%)/0 (0%)	1 (20%)/1 (5%)	0 (0%)/2 (10%)	0 (0%)/0 (0%)	0 (0%)/0 (0%)
2	0.6 mg m^−2^ × 5 days	3	10	0 (0%)/0 (0%)	1 (33%)/1 (10%)	0 (0%)/0 (0%)	2 (67%)/4 (40%)	1 (33%)/1 (10%)	0 (0%)/0 (0%)
3	0.6 mg m^−2^ × 3 days	6	17	0 (0%)/0 (0%)	0 (0%)/0 (0%)	0 (0%)/6 (35%)	2 (33%)/4 (24%)	0 (0%)/0 (0%)	0 (0%)/0 (0%)
4	0.8 mg m^−2^ × 3 days	3	22	0 (0%)/1 (5%)	0 (0%)/0 (0%)	0 (0%)/6 (27%)	1 (33%)/7 (32%)	0 (0%)/0 (0%)	0 (0%)/0 (0%)
5	1.0 mg m^−2^ × 3 days	7	32	2 (29%)/4 (13%)	0 (0%)/0 (0%)	2 (29%)/8 (25%)	4 (57%)/9 (28%)	0 (0%)/0 (0%)	0 (0%)/0 (0%)
6	1.2 mg m^−2^ × 3 days	8	25	1 (13%)/2 (8%)	0 (0%)/0 (0%)	0 (0%)/4 (16%)	7 (88%)/19 (76%)	1 (13%)/2 (8%)	0 (0%)/0 (0%)
7	1.4 mg m^−2^ × 3 days	4	18	0 (0%)/0 (0%)	0 (0%)/0 (0%)	0 (0%)/4 (22%)	4 (100%)/14 (78%)	0 (0%)/1 (6%)	0 (0%)/0 (0%)

a Number (percentage) of patients developing toxicity in the first course/number (percentage) of courses causing toxicity.

). To investigate further dose-intensification topotecan dosing was reduced from 5 to 3 days, yielding a total dose at level 3 (1.8 mg topotecan) similar to level 1 (2.0 mg topotecan). At dose level 3, six patients were included, because the first three only had five evaluable courses due to progressive disease. At dose level 6, two out of eight patients experienced DLT meeting the MTD criteria of ⩾two out of six patients, and it was concluded that the MTD was achieved at dose level 6 (topotecan 1.2 mg m^−2^ day^−1^ × 3 and IF 1.2 g m^−2^ day^−1^ × 3). Four additional patients were entered at dose level 5 at the recommended dose.

### Haematological toxicity

Myelosuppression was the principal toxicity. Table 3 lists the frequency of the grade III and IV haematological toxicities during treatment course 1 and during all courses. A total of 144 courses were evaluated, involving all 36 patients. Overall, grade III thrombocytopenia (3%) and grade III (6%) and IV (1%) anaemia were rare. The single observed grade IV anaemia was considered not related to the study drugs. Neutropenia grades III and IV occurred in 29 (20%) and 59 (41%) of all courses, respectively. Neutropenic fever was observed in seven (5%) courses. In all, 23 (16%) of all treatment courses were delayed for 1 week. Three patients had unresolved neutropenia at day 21, one patient had two episodes of ileus, one patient had two episodes of severe ascitis, and three courses were postponed upon patients’ request. Dose reductions due to neutropenia were performed in 18 (13%) of 144 treatment courses involving nine patients. At dose level 2 (0.6 mg m^−2^ day^−1^ × 5), one patient received two courses at 75% of the intended dose and another patient received four courses with topotecan for 3 days instead of 5 days. At dose level 3, one patient received one course at 75% of the intended topotecan dose. At dose level 6, one patient developed grade IV neutropenia with fever, which was resolved before the start of the second course. However, the fever reoccurred during the second day of the second course. This prompted an early infusion discontinuation after the first day of the second course. One patient at dose level 6 experienced grade III thrombocytopenia and grade IV neutropenia with neutropenic fever and another patient at dose level 6 and two at dose level 7 experienced grade IV neutropenia >7 days. Consequently, the dose of topotecan and IF was reduced by 25% in all subsequent courses in these patients.

There was no indication of cumulative myelosuppression on repeated dosing, evidenced by similar or less prominent haemoglobin, neutrophil, and platelet nadirs in later courses compared with the first course (Table 4

**Table 4:** Nadir blood counts in 144 courses

				**ANC (× 10^9^ l^−1^)**		**Haemoglobin (mmol l^−1^)**		**Platelets (× 10^9^ l^−1^)**	
				**1st course**	**All courses**	**1st course**	**All courses**	**1st course**	**All courses**
**Dose level**	**Topotecan dose**	**Number of patients**	**Number of courses**	**Median (range)**	**Median (range)**	**Median (range)**	**Median (range)**	**Median (range)**	**Median (range)**
1	0.4 mg m^−2^ × 5 days	5	20	1.9 (0.7–3.5)	2.0 (0.4–3.5)	6.4 (5.4–7.1)	6.5 (4.4–7.1)	230 (99–403)	165 (99–445)
2	0.6 mg m^−2^ × 5 days	3	10	0.4 (0.2–1.3)	1.0 (0.2–2.4)	5.6 (3.4–5.7)	5.7 (3.4–6.6)	79 (25–231)	212 (25–282)
3	0.6 mg m^−2^ × 3 days	6	17	1.4 (0.1–3.7)	0.7 (0.04–4.0)	6.2 (5.9–7.0)	5.9 (5.0–7.3)	190 (125–283)	175 (78–283)
4	0.8 mg m^−2^ × 3 days	3	22	2.1 (0.3–3.5)	0.7 (0.1–3.5)	6.1 (5.7–7.5)	6.5 (4.7–7.5)	176 (129–230)	197 (129–279)
5	1.0 mg m^−2^ × 3 days	7	32	0.5 (0.1–1.1)	0.8 (0.1–2.3)	5.5 (4.0–6.8)	5.7 (4.0–7.1)	193 (115–269)	175 (110–514)
6	1.2 mg m^−2^ × 3 days	8	25	0.2 (0.02–2.1)	0.3 (0.02–2.1)	6.2 (4.9–7.0)	6.1 (4.8–7.1)	101 (44–201)	131 (37–419)
7	1.4 mg m^−2^ × 3 days	4	18	0.3 (0.05–0.3)	0.3 (0.05–0.6)	7.1 (5.3–7.8)	6.3 (5.2–7.8)	112 (66–237)	150 (38–279)

ANC=absolute neutrophil count.

). The neutrophil nadir typically occurred between days 7 and 14 (median 10 days). Absolute neutrophil counts at dose level 2 (dose intensity 3.0 mg m^−2^) were comparable to dose levels 5 and 6 (dose intensities 3.0 and 3.6 mg m^−2^, respectively). A similar pattern was observed with the haemoglobin and platelet levels of dose levels 2 and 6. Most patients in the study received infusions of packed cells due to anaemia.

### Nonhaematological toxicity

Nonhaematological toxicities were relatively mild and not dose related (Table 5

**Table 5:** Nonhaematological toxicity in 144 courses of 36 patients

	**Percentage of courses (number of patients)**				**Percentage of courses (number of patients)**		
**Toxicity**	**Grade I**	**Grade II**	**Grade III**	**Toxicity**	**Grade I**	**Grade II**	**Grade III**
Vomiting	24% (16)	10% (8)	1% (1)	Peripheral neuropathy	14% (6)	2% (1)	
Nausea	35% (23)	20% (14)	3% (4)	Vertigo	3% (4)		
Abdominal cramps	4% (5)	2% (2)		Headache	5% (6)	2% (2)	
Constipation	30% (23)	8% (9)	1% (2)	Anxiety		5% (4)	
Anorexia		2% (3)		Pain	15% (9)	5% (6)	5% (5)
Diarrhoea	8% (10)	3% (3)	1% (1)	Myalgia	4% (5)	1% (1)	
Fatigue	15% (13)	17% (13)	2% (2)	Dyspnoea	1% (2)	3% (3)	2% (3)
Fever	7% (8)	5% (5)	3% (4)	Infection	3% (5)	4% (6)	
Asthenia	6% (4)	4% (3)	2% (3)	Cough	7% (7)		
Alopecia	9% (9)	47% (26)		Haemorrhage (urine)	1% (1)		

). One patient (six courses) with peripheral neuropathy grade I had pre-existing neuropathy due to cisplatin pretreatment. In one patient, an episode of sensory neuropathy grade II was suspected to be treatment related, but in another patient an episode of grade III was considered not to be related, because of confirmed CNS metastases. All toxicities, including neuropathy, were reversible and rapidly vanished after therapy was stopped.

Blood chemistry prior to treatment did not exceed reference values. The median creatinine CL prior to treatment was 92 ml min^−1^ and ranged between 48 and 148 ml min^−1^; this was unaltered prior to the second course. In one patient, increased levels of AP, ASAT, and ALAT (946, 103, and 132 U/l, respectively) were observed after the first course. Later, this patient was found to have an obstructed biliary tract due to tumour growth. Between courses 1 and 2, a total of six and 10 patients had calcium and magnesium levels below the reference values of 2.2 and 0.7 *μ*mol l^−1^, respectively.

### Pharmacokinetics

**Figure 1:**
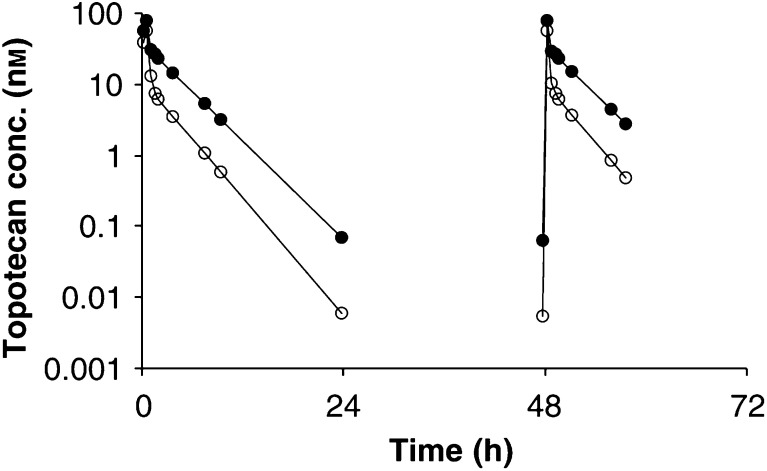
A typical plasma concentration–time profile of total topotecan (solid markers) and topotecan lactone (open markers) of a patient receiving a 30-min administration of 1.2 mg m^−2^ day^−1^ topotecan for 3 consecutive days.

Total topotecan pharmacokinetic data were obtained in the first course of 32 patients and in the second course of 17 patients, respectively. Lactone topotecan pharmacokinetic data were obtained in the first course of 16 patients and in the second course of 15 patients. Typical plasma profiles of topotecan lactone and total topotecan of a patient receiving 1.2 mg m^−2^ day^−1^ topotecan are displayed in Figure 1

. The lactone form was the predominant form during the infusion, but approximately 30 min after the end of the infusion the open-ring (carboxylate) form concentrations exceeded lactone levels. The mean (±s.d.) lactone-to-total topotecan ratio at the end of the infusion was 0.74±0.10 (*n*=23). After the end of the infusion, lactone curves exhibited a biexponential decay. The population pharmacokinetic parameters of topotecan lactone and total topotecan are listed in Table 6

**Table 6:** Pharmacokinetics of topotecan lactone and total topotecan (lactone and carboxylate form) with their s.e., IIV, IOV and RV

	**Topotecan lactone**				**Total topotecan**			
	**Estimate (±s.e.)**	**IIV**	**IOV**	**RV**	**Estimate (±s.e.)**	**IIV**	**IOV**	**RV**
CL (l h^−1^)	68.0±3.2	16%	9%		24.9±1.2	23%	12%	
*V* (l)	29.5±1.2	22%	—		16.6±3.5	45%	—	
*V*_ss_ (l)	133±7	16%	—		80.0±2.8	17%	—	
*Q* (l h^−1^)	66.3±2.3	4%	—		129±23	12%	—	
PE				15.9%				13.0%
AE (*μ*M)				0.150				0.392

CL=clearance, *V*=volume of distribution of central compartment, *V*_ss_=volume of distribution at steady state, *Q*=intercompartmental clearance, PE=proportional error, AE=additive error; s.e.=standard error; IIV=interindividual variability; IOV=interoccasion variability; RV=residual variability.

**Figure 2:**
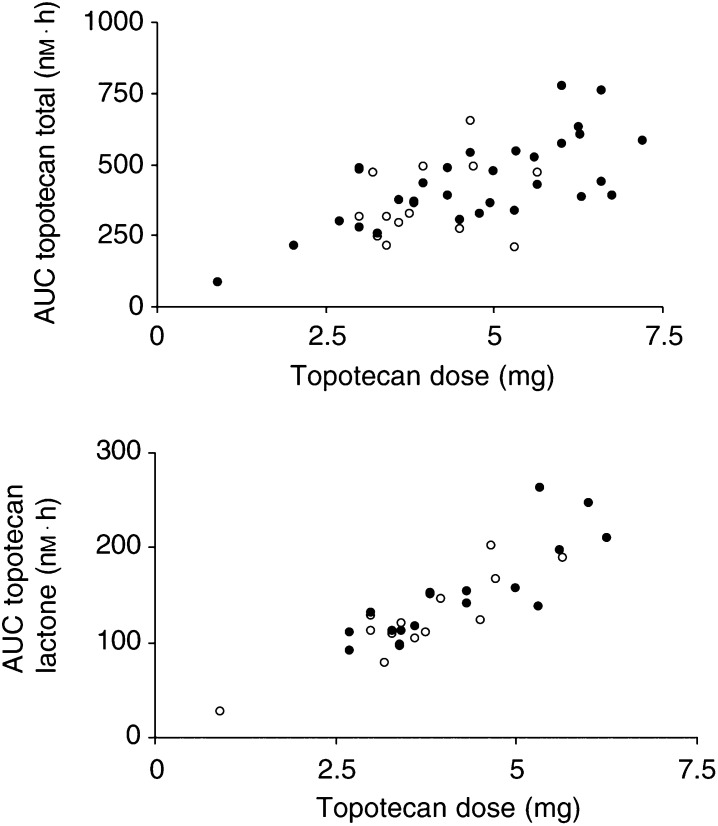
Relationship between the total topotecan dose (mg) per course and the area under the plasma concentration–time curve (AUC) of total topotecan (upper graph) and topotecan lactone (lower graph) in course 1 (solid marker) and 2 (open marker).

. Variability between patients and between courses 1 and 2 was modest. The relationship between the AUC of lactone and total topotecan with the absolute topotecan dose is represented in Figure 2

. Moderate IIV in exposure was observed.

**Figure 3:**
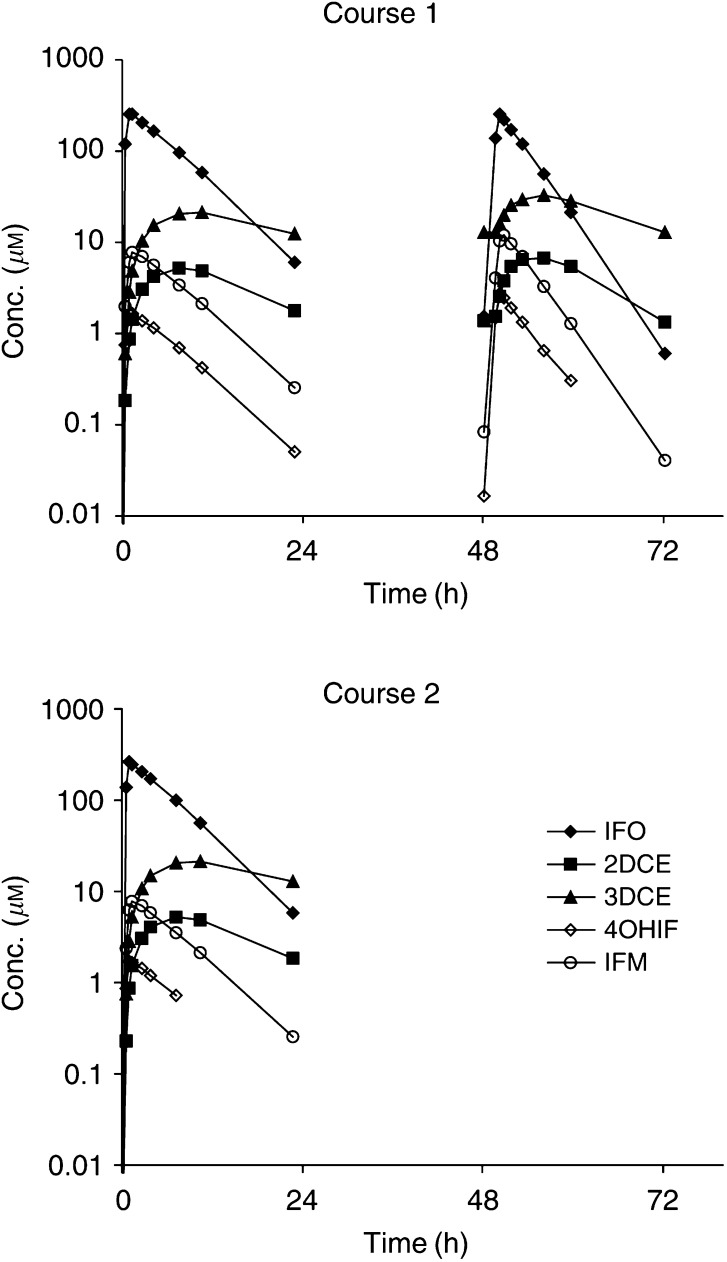
Typical plasma concentration–time profiles of IF, 2DCE, 3DCE, 4OHIF, and IFM of a patient receiving a 1-h infusion of 1.2 g m^−2^ day^−1^ ifosfamide for 3 consecutive days with a 3-week interval.

Plasma concentration–time profiles of IF and its metabolites are presented in Figure 3

. A decrease in the elimination half-life of IF was observed when comparing the monoexponential decays on days 1 and 3, due to the autoinduction of the IF metabolism. This effect was reset at the start of the next course. The population pharmacokinetic parameters of IF and its metabolites are listed in Table 7

**Table 7:** Pharmacokinetics of IF, 2DCE, 3DCE, 4OHIF, and IFM with their s.e., IIV, IOV, and RV

	**Ifosfamide**					**Deactivated metabolites**				**Activated metabolites**		
	**Estimate (±s.e.)**	**IIV (%)**	**IOV**	**RV**		**Estimate (±s.e.)**	**IIV (%)**	**RV**		**Estimate (±s.e.)**	**IIV (%)**	**RV**
CL_init_ (l h^−1^)	3.52±0.18	22	6%		F^*^_2DCE_ (l^−1^)	0.00406±0.00077	70		F^*^_4OHIF_ (l^−1^)	0.147±0.013	43	
*V*_IFO_ (l)	32.3±1.0	14	—		*K*_2DCE_ (h^−1^)	0.118±0.021	27		K_4OHIF_ (h^−1^)	73.8	—	
*K*_enz, out_ (h^−1^)	0.0137±0.0012	20	—		AUC_2DCE_ (*μ*M h)	0.861±0.132			AUC_4OHIF_ (*μ*M h)	0.0482±0.0044		
t_1/2,enz(h)_	50.6				PE			0%	PE			23.0%
AUC_IFO_ (*μ*M h)	4.67±0.27				AE (*μ*M)			5.10	AE (*μ*M)			0.274
PE				14.3%	F^*^_3DCE_ (l^−1^)	0.00633±0.00044	29		F^**^_IFM_ (h^−1^)	0.56±0.36	—	
AE (*μ*M)				4.51	*K*_3DCE_ (h^−1^)	0.0752±0.0038	—		*K*_IFM_ (h^−1^)	4.0±1.5	76	
					AUC_3DCE_ (*μ*M h)	2.04±0.13			AUC_IFM_ (*μ*M h)	0.399±0.089		
					PE			17.3%	PE			10.7%
					AE (*μ*M)			3.22	AE (*μ*M)			4.50

CL_init_=initial ifosfamide clearance; *V*_IFO_=volume of distribution of central compartment of ifosfamide; *K*_enz,out_=first-order rate constant for enzyme degradation/inactivation; *t*_1/2,enz_ (h)=induction half-life of the enzyme; AUC=area under the plasma concentration–time curve during the first course; PE=proportional error; AE=additive error; F^*^=ratio of fraction metabolised and volume of distribution of metabolite; *K*=first-order rate constant for metabolite elimination; F^**^=formation rate constant; IF=ifosfamide; 2DCE=2-dechloroethylifosfamide; 3DCE=3-dechloroethylifosfamide; 4OHIF=4-hydroxyifosfamide; IFM=ifosforamide mustard; s.e.=standard error; IIV=interindividual variability; IOV=interoccasion variability; RV=residual variability.

. The concentration–time data did not allow estimation of IC_50_ and it was therefore fixed at 0.001 *μ*M, which was below the lower limit of quantification. Fixing IC_50_ resulted in an adequate description of the development of autoinduction of the IF metabolism. Insufficient data were available to estimate the elimination rate of the hydroxylated metabolite (*K*_4OHIF_), which was therefore fixed to a value (73.8 h^−1^) obtained from a prior analysis (Kerbusch *et al*, 2001b). Variability between patients in IF pharmacokinetics was modest and somewhat larger for the metabolites. The mean (±s.d.) urinary excretion of IF was 34.7±12.1% of the administered dose with 17.8±10.0% as unchanged IF, 5.6±2.2% as 2DCE, and 11.2±3.8% as 3DCE.

### Pharmacodynamics

**Figure 4:**
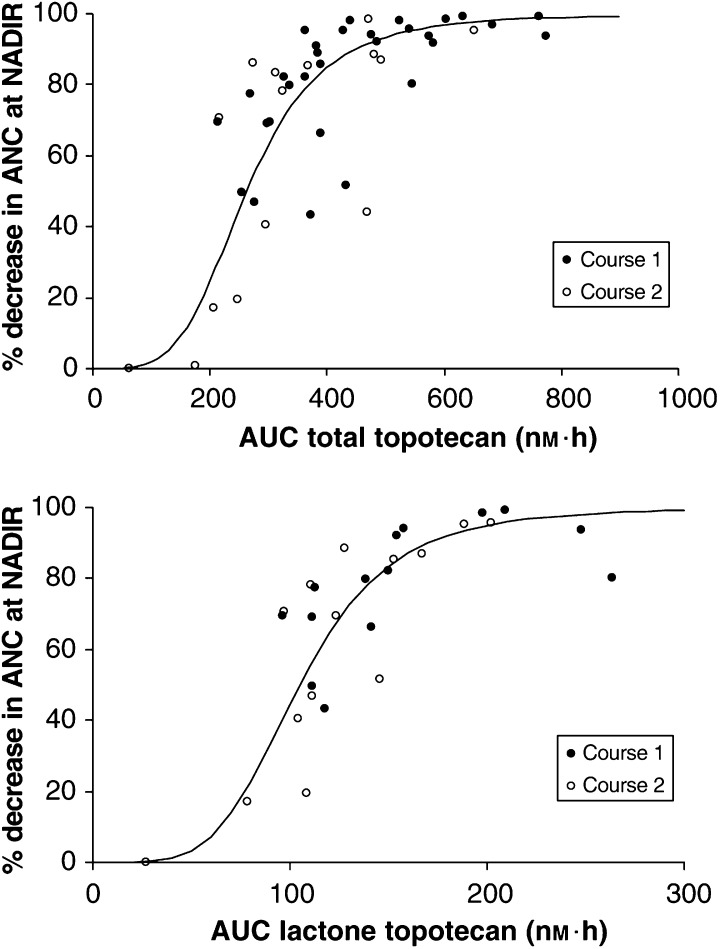
Percentage decrease in absolute neutrophil count (ANC) *vs* the area under plasma concentration–time curve (AUC) of total (upper graph) and lactone (lower graph) topotecan during course 1 (solid markers) and course 2 (open markers). The line indicates the best fit of the data to the sigmoidal–*E*_max_ model.

The relationships between the AUC for the lactone and total topotecan and the percentage decrease in neutrophils could adequately be described by sigmoidal–*E*_max_ models (Figure 4

). Estimates for AUC_50_ and the Hill coefficient are presented in Table 8

**Table 8:** Estimated parameters with their s.e. of the relationship between the area under the plasma concentration–time curve (AUC) of lactone and total topotecan and the percentage decrease in ANC and THRs in courses 1 and 2 of 23 patients

	**Topotecan lactone**		**Total topotecan**	
	**Estimate (±s.e.)**	**IOV**	**Estimate (±s.e.)**	**IOV**
*Decrease in ANC*				
*E*_max_ (%)	100 fixed		100 fixed	
AUC_50_ (*μ*M)	105±8	22%	263±23	29%
*γ*	4.54±1.14		4.11±0.66	
AE	0.4%		0.1%	
				
*Decrease in thrombocytes*				
*E*_max_ (%)	100 fixed		100 fixed	
AUC_50_ (*μ*M)	131±11	41%	376±41	64%
*γ*	1.77±0.58		1.09±0.36	
AE	—		8.9%	

IOV=interoccasion variability (random variability between courses 1 and 2); *E*_max_=maximum effect; AUC_50_=topotecan AUC associated with 50% of *E*_max_; *γ*=Hill coefficient; AE=additive error; s.e.=standard error; ANC=absolute neutrophil count; THR=thrombocyte; AE=additive error.

. The individual estimates of the AUC_50_ between courses 1 and 2 decreased with 9 and 12%, respectively. However, this difference in potency was not statistically significant (*P*<0.05), because including a nonrandom course effect only resulted in a drop of the objective function of 1. Differences between courses 1 and 2 were better described with random variability in AUC_50_. The high Hill coefficients (>4) indicated a steep dose–response relationship for both compounds. Adding 4OHIF exposure to the model did not result in an improvement of goodness-of-fit of this relationship. Various nested models were tested where the Hill coefficients and AUC_50_’s of the active moieties were assumed equal. None of the models achieved a successful description of the additivity.

**Figure 5:**
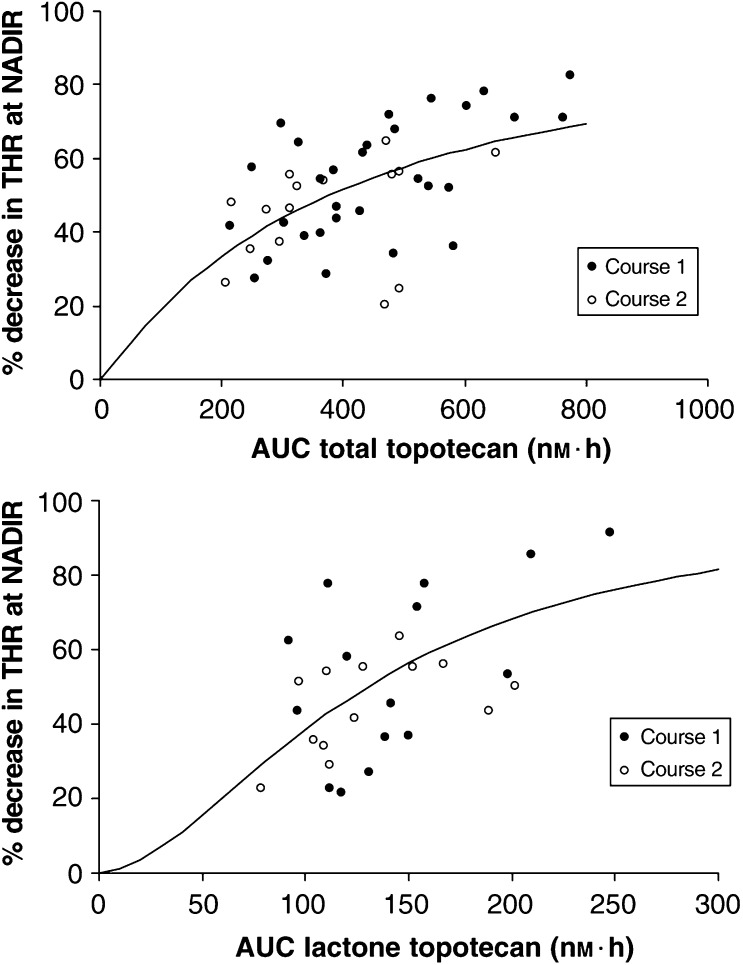
Percentage decrease in thrombocytes (THR) *vs* the area under plasma concentration–time curve (AUC) of total (upper graph) and lactone (lower graph) topotecan during course 1 (solid markers) and course 2 (open markers). The line indicates the best fit of the data to the sigmoidal–*E*_max_ model.

The relationships between the AUC for the lactone and total topotecan and the percentage decrease in THR could also be described by sigmoidal–*E*_max_ models (Figure 5

). The relatively high standard errors of the estimates indicated lack of goodness-of-fit, probably due to a lack of information on either end of the extremes of the curve. Estimates of AUC_50_ and the Hill coefficient are also presented in Table 8. The RV and IIV were confounded due to insufficient data for the lactone form topotecan–THR relationship. Therefore, the RV could not be estimated.

### Responses

In all, 34 patients were assessable for therapeutic activity. Of these, 22 patients demonstrated stable disease and nine progressive disease during treatment. Partial responses were documented in three heavily pretreated patients all with ovarian cancer, which was the predominant primary tumour site (61%) in this study. One partial responder was dosed at the recommended dose and the other two at the two underlying three-daily dose levels.

## DISCUSSION

There are few anticancer agents that have significant therapeutic impact when used alone, particularly in the treatment of solid tumours. The single-agent and combination therapy activities of topotecan and IF have been extensively studied (Herben *et al*, 1996; Kerbusch *et al*, 2001a). Murren *et al* (1997) proposed a synergistic cytotoxicity between topoisomerase-I inhibitors and alkylating agents. Bioactivation of IF leads to the alkylating compound IFM, which possesses a high affinity for macromolecules such as DNA. DNA alkylation leads to crosslink formation finally resulting in cytotoxicity and cell death through apoptosis. DNA alkylation initiates DNA repair mechanisms. Topoisomerase-I may be involved in this machinery through unwinding of the DNA. However, its direct relationship to the cytotoxic events of IF is at present unclear. The suggested synergism encouraged us to evaluate the feasibility and safety profile of the combination topotecan with IF in cancer patients.

Haematological toxicity, especially neutropenia was the DLT for this combination. Topotecan could not be safely administered in the approved single agent (1.5 mg m^−2^) daily times five schedule. Severe neutropenia and thrombocytopenia occurred at a dose of 0.6 mg m^−2^ day^−1^ for 5 days. To investigate further dose intensification, topotecan dosing was reduced from 5 to 3 days. With the adjusted schedule, topotecan was escalated to 1.4 mg m^−2^ day^−1^. At that dose level DLT was observed. Additional patients at one dose level below the highest also demonstrated unacceptable toxicity. Therefore, the recommended dose was found to be 1.0 mg m^−2^ day^−1^ topotecan × 3 days and 1.2 g m^−2^ day^−1^ IF × 3 days. Seven patients were treated at the recommended dose with acceptable toxicity.

The neutropenia was correlated with the dose intensity of topotecan (Table 3), which was also observed in the previous studies (van Warmerdam *et al*, 1995). Neutropenia is the most frequently observed toxicity associated with single-agent topotecan. In phase II trials of single-agent topotecan in adult patients with solid tumours receiving 30-min infusions of 1.5–2.0 mg m^−2^ day^−1^ for 5 days every 3 or 4 weeks, grade III–IV neutropenia was observed in 71–75% of all courses (Creemers *et al*, 1995; Perez-Soler *et al*, 1996). The haematological toxicity was generally of short duration, noncumulative, and manageable. Ifosfamide's principal toxicity is also haematological, albeit less severe than of its isomer cyclophosphamide. A WBC count <3 × 10^9^ l^−1^ is expected in approximately 50% of patients and platelet counts <100 × 10^9^ l^−1^ in 20% of patients treated with single-agent IF at a dose of 1.2 g m^−2^ day^−1^ for 5 days (Telekes and Hendrich, 1996). Their overlapping toxicity profiles prevented escalation of topotecan, in combination with IF, to a dose near the single-agent MTD of 1.5 mg m^−2^ day^−1^ for 5 consecutive days.

Eight episodes (6%) of grade III anaemia were observed in all courses, which were considered related to the study drugs. This is in accordance with the observation that significant anaemia occurred in 2–14% of all courses of phase II studies in patients receiving single-agent topotecan at 1.5–2.0 mg m^−2^ day^−1^ for 5 days (Creemers *et al*, 1995; Perez-Soler *et al*, 1996).

Only four episodes (3%) of grade III thrombocytopenia were observed in our study. Thrombocytopenia associated with single-agent topotecan is rare and mainly reported after prolonged infusion schedules (Herben *et al*, 1998). Severe thrombocytopenia was observed in 2–10% of all courses in the above-mentioned phase II studies of the daily × 5 schedule (Creemers *et al*, 1995; Perez-Soler *et al*, 1996).

Bone marrow G-CSF was not allowed in this study. Granulocyte colony-stimulating factor could potentially improve the tolerance to the combination regimen. However, preliminary results presented by Bienvenu *et al* (2000) suggest the impact of G-CSF to be limited. They recommended a dose of 1.0 mg m^−2^ day^−1^ topotecan in combination with 1.5 g m^−2^ day^−1^ IF, both daily for 3 consecutive days with G-CSF support from days 5 to 12.

Recently, Schneider *et al* (2002) published a study of this combination therapy with G-CSF (when indicated) based on a 5-day schedule. They observed pronounced nonhaematological toxicity (hepatic and renal) and two treatment-related deaths out of 11 patients. This is in contrast to our findings. No hepatic toxicity, renal tubular abnormalities, or haemorrhagic cystitis were observed. All nonhaematological toxicities were relatively mild, not dose related, and transient with nausea, vomiting, fatigue, constipation, and alopecia being the most observed. These have been described before in phase II studies of single-agent topotecan with similar incidence rates (Creemers *et al*, 1995). Peripheral neuropathy or paresthesia has occurred rarely with topotecan treatment and a causal relationship is uncertain. The relatively high incidence of neuropathy in our study may be related to prior treatment with paclitaxel in three and cisplatin in one of five patients (Rowinsky *et al*, 1992). No encephalopathy, a specific IF-related toxicity, was observed in this study (Cerny and Küpfer, 1992).

Population pharmacokinetic parameters of topotecan were similar to previously reported parameter estimates obtained using a population approach (Gallo *et al*, 2000) and a standard two-stage analysis (Wall *et al*, 1992; van Warmerdam *et al*, 1995) after single-agent administration of 0.5–2.0 mg m^−2^ topotecan in the daily times five schedule. Gallo *et al* reported a CL of 32 l h^−1^ and volume of distribution at steady state (*V*_ss_) of 120 l, whereas the current analysis found 29 l h^−1^ and 80 l, respectively. This minor discrepancy may be explained by differences in the characteristics of the studied patients. Gallo *et al* identified weight, height serum creatinine, and sex as significant covariates for CL and *V*_ss_. The median values/frequency of these covariates were lower in the current study resulting in lower estimates of CL and *V*_ss_. The CL of the lactone (68 l h^−1^) form exceeded the total form (29 l h^−1^). This apparent CL of the lactone form is actually CL/F with F being the fraction of the total concentration/amount of dose in lactone form. Similar reasoning can be followed for *V*, *V*_ss_ and *Q*. Metabolic interactions of topotecan with concomitantly administered IF were not anticipated, because topotecan metabolism to *N*-desmethyltopotecan is only 0.4–3% of the dose (Herben *et al*, 1998).

Nephrotoxicity is frequently encountered with IF treatment. Since approximately 40% of topotecan is eliminated by the kidneys, IF-induced renal tubular damage could potentially alter the renal CL of topotecan. In the current study, no episodes of renal toxicity were observed. Creatinine CL, an indicator of renal function, did not exceed reference values. In agreement with these clinical findings, plasma profiles of topotecan on days 2 and 3 were similar to those on day 1, which indicates that IF coadministration at the applied dose regimen most likely did not interfere with topotecan CL.

Population pharmacokinetic parameters of IF and metabolites were similar to those reported in a previous pharmacokinetic study using an identical model (Kerbusch *et al*, 2001b). In another study, high doses of IF (9–12 g m^−2^) were administered as single agent by a 72-h continuous infusion (Kerbusch *et al*, 2000b). A four-fold higher value for *t*_1/2,enz_ was obtained in the current study. Furthermore, no IC_50_ could be estimated in the current study. This may be explained by the fact that estimation of autoinduction in a pharmacokinetic profile of a 1-h infusion is inherently less feasible than in that of a 72-h continuous infusion of IF. Although the combination was never administered in reverse order, topotecan and IF did not appear to interact pharmacokinetically in the studied combination regimen, since the observed pharmacokinetics were similar to those after single-agent administration.

The estimates for the parameters used to describe the sigmoidal–*E*_max_ relationship of the pharmacokinetics and the myelosuppression were similar to previously reported values. van Warmerdam *et al* (1995) reported an AUC_50_ for total topotecan of 173 nM h^−1^ after single-agent topotecan (0.5–1.5 mg m^−2^ day^−1^ × 5). In contrast, the Hill coefficients were two-fold higher than previously reported (1.8), possibly indicating the additive effect of IF on the myelosuppression. The relationship between the topotecan exposure and the decrease in THR indicated that the maximum decrease is not likely to be reached with the doses studied. No structural differences (only random) were observed in the estimates for the AUC_50_ in the first and second course. This was in accordance with the observation that neutropenia was not cumulative. So far, dose escalation of topotecan only reached approximately 50% of the MTD of single-agent treatment with topotecan. This indicated a considerable additive myelosuppressive effect of IF. Exposure to the activated metabolites of IF (4OHIF and IFM) ranged approximately five-fold in the study, although the IF dose was not escalated. However, no clear relationship between the exposure to these active metabolites and the myelosuppression was observed (data not shown). Additive modelling of topotecan lactone/total and 4OHIF did not result in an increased goodness-of-fit of the relationship with myelosuppression. The inability to detect this relationship can be explained by the limited number of subjects, in which all data were available (*n*=14) and the lack of an IF dose escalation or myelosuppresion data after single-agent topotecan or IF administration.

This phase I dose-escalating study was not designed for an evaluation of efficacy. Nonetheless, 8% of the patients refractory to standard therapy did demonstrate clinical benefit (partial response) from this combination with dosing at and below the recommended dose.

It was concluded that the combination treatment of topotecan at 1.0 mg m^−2^ day^−1^ × 3 days with IF at 1.2 g m^−2^ day^−1^ × 3 days was feasible. Possible clinical benefit and synergism of this combination can only be evaluated in a phase II trials. However, the combination schedule of topotecan and IF did result in considerable haematological toxicity and in conjunction with previously reported pronounced nonhaematological toxicities and treatment-related deaths, it may be concluded that this is not a favourable combination. The combined tolerated dose was lower than expected based on single-agent experiences. No pharmacokinetic interaction could be detected. It is therefore likely that the combination is characterised by overlapping toxicity.
